# Supplementary material for: Alternative c-MYC mRNA Transcripts as an Additional Tool for c-Myc2 and c-MycS Production in BL60 Tumors
Source: Biomolecules. 2022 Jun 16;12(6):836. doi: 10.3390/biom12060836 (PMC9221284; doi:10.3390/biom12060836)
Supplement: Supplementary file 1 [file biomolecules-12-00836-s001.zip › biomolecules-1710344-supplementary.pdf]

Supplementary figure legends

```
C-Myc67   MDFFRVVENQPPATMPLNVSFTNRNYDLDYDSVQPYFYCDEEENFYQQQQQSELQPPAPS 60
C-Myc64   --------------MPLNVSFTNRNYDLDYDSVQPYFYCDEEENFYQQQQQSELQPPAPS 46
C-MycS    ------------------------------------------------------------ 0
C-MycS1   ------------------------------------------------------------ 0
C-MycS2   ------------------------------------------------------------ 0

C-Myc67   EDIWKKFELLPTPPLSPSRRSGLCSPSYVAVTPFSLRGDNDGGGGSFSTADQLEMVTELL 120
C-Myc64   EDIWKKFELLPTPPLSPSRRSGLCSPSYVAVTPFSLRGDNDGGGGSFSTADQLEMVTELL 106
C-MycS    ------------------------------------------------------MVTELL 6
C-MycS1   ------------------------------------------------------------ 0
C-MycS2   ------------------------------------------------------------ 0

C-Myc67   GGDMVNQSFICDPDDETFIKNIIIQDCMWSGFSAAAKLVSEKLASYQAARKDSGSPNPAR 180
C-Myc64   GGDMVNQSFICDPDDETFIKNIIIQDCMWSGFSAAAKLVSEKLASYQAARKDSGSPNPAR 166
C-MycS    GGDMVNQSFICDPDDETFIKNIIIQDCMWSGFSAAAKLVSEKLASYQAARKDSGSPNPAR 66
C-MycS1   ---MVNQSFICDPDDETFIKNIIIQDCMWSGFSAAAKLVSEKLASYQAARKDSGSPNPAR 57
C-MycS2   ---------------------------MWSGFSAAAKLVSEKLASYQAARKDSGSPNPAR 33
                                     *********************************

C-Myc67   GHSVCSTSSLYLQDLSAAASECIDPSVVFPYPLNDSSSPKSCASQDSSAFSPSSDSLLSS 240
C-Myc64   GHSVCSTSSLYLQDLSAAASECIDPSVVFPYPLNDSSSPKSCASQDSSAFSPSSDSLLSS 226
C-MycS    GHSVCSTSSLYLQDLSAAASECIDPSVVFPYPLNDSSSPKSCASQDSSAFSPSSDSLLSS 126
C-MycS1   GHSVCSTSSLYLQDLSAAASECIDPSVVFPYPLNDSSSPKSCASQDSSAFSPSSDSLLSS 117
C-MycS2   GHSVCSTSSLYLQDLSAAASECIDPSVVFPYPLNDSSSPKSCASQDSSAFSPSSDSLLSS 93
          ************************************************************

C-Myc67   TESSPQGSPEPLVLHEETPPTTSSDSEEEQEDEEEIDVVSVEKRQAPGKRSESGSPSAGG 300
C-Myc64   TESSPQGSPEPLVLHEETPPTTSSDSEEEQEDEEEIDVVSVEKRQAPGKRSESGSPSAGG 286
C-MycS    TESSPQGSPEPLVLHEETPPTTSSDSEEEQEDEEEIDVVSVEKRQAPGKRSESGSPSAGG 186
C-MycS1   TESSPQGSPEPLVLHEETPPTTSSDSEEEQEDEEEIDVVSVEKRQAPGKRSESGSPSAGG 177
C-MycS2   TESSPQGSPEPLVLHEETPPTTSSDSEEEQEDEEEIDVVSVEKRQAPGKRSESGSPSAGG 153
          ************************************************************

C-Myc67   HSKPPHSPLVLKRCHVSTHQHNYAAPPSTRKDYPAAKRVKLDSVRVLRQISNNRKCTSPR 360
C-Myc64   HSKPPHSPLVLKRCHVSTHQHNYAAPPSTRKDYPAAKRVKLDSVRVLRQISNNRKCTSPR 346
C-MycS    HSKPPHSPLVLKRCHVSTHQHNYAAPPSTRKDYPAAKRVKLDSVRVLRQISNNRKCTSPR 246
C-MycS1   HSKPPHSPLVLKRCHVSTHQHNYAAPPSTRKDYPAAKRVKLDSVRVLRQISNNRKCTSPR 237
C-MycS2   HSKPPHSPLVLKRCHVSTHQHNYAAPPSTRKDYPAAKRVKLDSVRVLRQISNNRKCTSPR 213
          ************************************************************

C-Myc67   SSDTEENVKRRTHNVLERQRRNELKRSFFALRDQIPELENNEKAPKVVILKKATAYILSV 420
C-Myc64   SSDTEENVKRRTHNVLERQRRNELKRSFFALRDQIPELENNEKAPKVVILKKATAYILSV 406
C-MycS    SSDTEENVKRRTHNVLERQRRNELKRSFFALRDQIPELENNEKAPKVVILKKATAYILSV 306
C-MycS1   SSDTEENVKRRTHNVLERQRRNELKRSFFALRDQIPELENNEKAPKVVILKKATAYILSV 297
C-MycS2   SSDTEENVKRRTHNVLERQRRNELKRSFFALRDQIPELENNEKAPKVVILKKATAYILSV 273
          ************************************************************

C-Myc67   QAEEQKLISEEDLLRKRREQLKHKLEQLRNSCA 453
C-Myc64   QAEEQKLISEEDLLRKRREQLKHKLEQLRNSCA 439
C-MycS    QAEEQKLISEEDLLRKRREQLKHKLEQLRNSCA 339
C-MycS1   QAEEQKLISEEDLLRKRREQLKHKLEQLRNSCA 330
C-MycS2   QAEEQKLISEEDLLRKRREQLKHKLEQLRNSCA 306
          *********************************
```

Figure S1: Alignment of the predicted primary translational structures of the five open reading frames corresponding to c-Myc1, c-Myc2 c-MycS previously described plus two additional potential open reading frames c-MycS1 and cMycS2 suggested by the present work. Note that all these isoforms differ only in the N-terminal part.

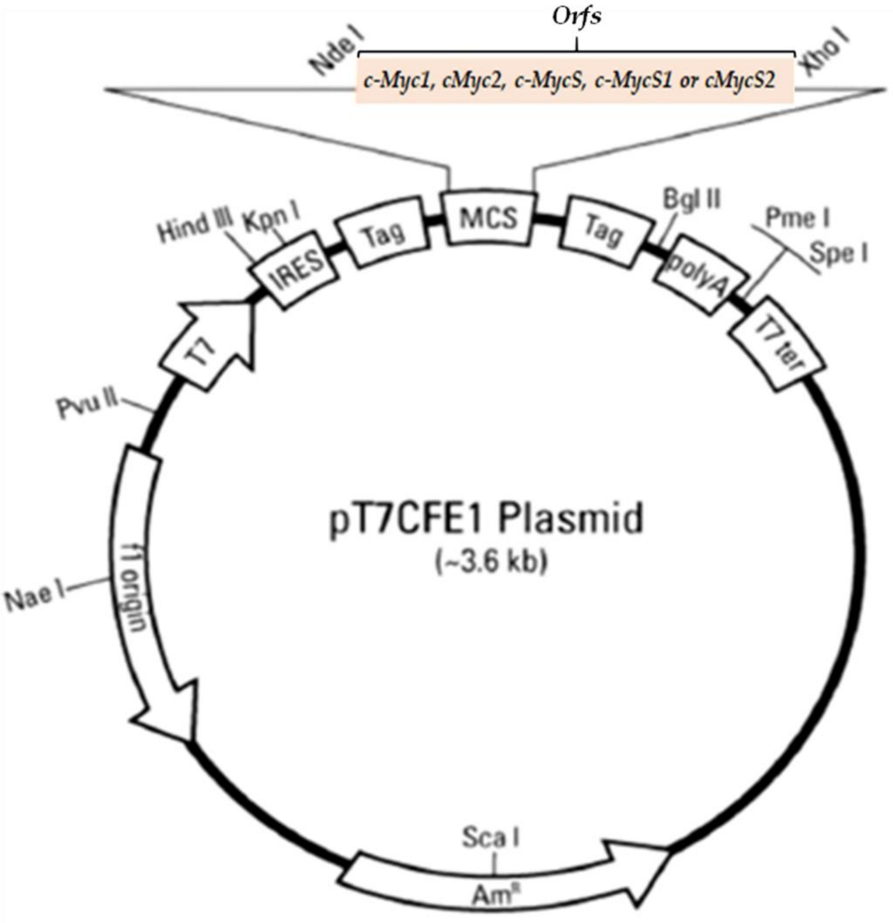

<u>Figure S2</u>: Full-length open reading frames (Orf) encoding c-Myc1, c-Myc2 or c-MycS, c-MycS1 and c-MycS2 cloned into the pT7CFE1 vector subjected to transcription (T7 promoter) and translation using a mammalian in vitro translation system based on HeLa cell lysates

```
Genomic  TAAGGAAAACGATTCCTTCTAACAGAAATGTCCTGAGCAATCACCTATGAACTTGTTTCAAATGCATGATCAAATGCAACCTCACAACCTTGGCTGAGTCTTGAGACTGAAAGATTTAGC 120
3'UTR1   TAAGGAAAACGATTCCTTCTAACAGAAATGTCCTGAGCAATCACCTATGAACTTGTTTCAAATGCATGATCAAATGCAACCTCACAACCTTGGCTGAGTCTTGAGACTGAAAGATTTAGC
3'UTR2   TAAGGAAAACGATTCCTTCTAACAGAAATGTCCTGAGCAATCACCTATGAACTTGTTTCAAATGCATGATCAAATGCAACCTCACAACCTTGGCTGAGTCTTGAGACTGAAAGATTTAGC

         ------------------------------------------/continuation of 3'UTR/------------------------------------------

Genomic  TACACAATGTTTCTCTGTAAATATTGCCATTAAATGTAAATAACTTTAATAAAACGTTTATAGCAGTTACACAGAATTTCAATCCTAGTATATAGTACCTAGTATTATAGGTACTATAAA 360
3'UTR1   TACACAATGTTTCTCTGTAAATATTGCCATTAAATGTAAATAACTTTAATAAAACGTTTATAGCAGTTAAAAAAAAAAAAAAAAAAAAAAAAAAA ——► Poly A tail
3'UTR2   TACACAATGTTTCTCTGTAAATATTGCCATTAAATGTAAATAACTTTAATAAAACGTTTATAGCAGTTACACAGAATTTCAATCCTAGTATATAGTACCTAGTATTATAGGTACTATAAA

Genomic  CCCTAATTTTTTTTATTTAAGTACATTTTGCTTTTTAAAGTTGATTTTTTTCTATTGTTTTTAGAAAAAATAAAATAACTGGCAAATATATCATTGAGCCAAATCTTAAGTTGTGAATGT 480
3'UTR2   CCCTAATTTTTTTTATTTAAGTACATTTTGCTTTTTAAAGTTGATTTTTTTCTATTGTTTTTAGAAAAAATAAAATAACTGGCAAATATATCATTGAGCCAAATCTTAAAAAAAAAAAAAA ——► Poly A tail
```

<u>Figure S3</u>: Nucleotide sequences of the identified 3'UTR types. The translational TAA stop codon is indicated. Alternative AATAAA polyadenylation sites are underlined.

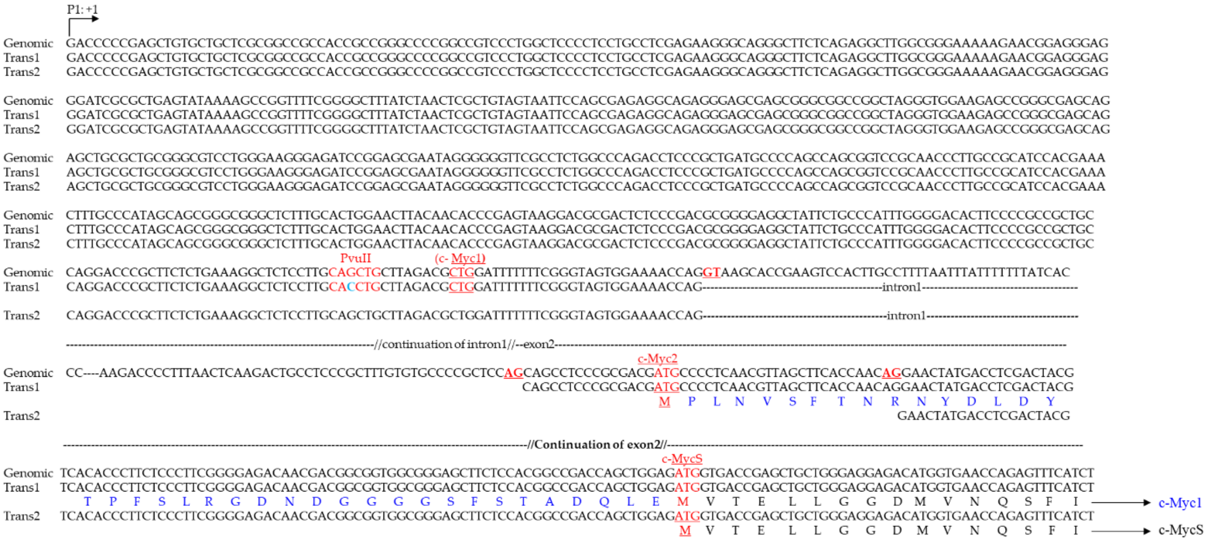

Figure S4: Nucleotide sequences of Trans1 and Trans2 transcripts compared to the corresponding genomic structure. Underlined splice donor (GT), acceptor (AG) sites, and initiation codons (CTG, ATG) are indicated. The first amino acids of c- Myc2 and c-MycS are shown. Translation from CTG of Trans1 is prevented by the PvuII mutation. P1:+1: Transcription initiation site from the P1 promoter. PFull1: Primer (Table1); M: The first amino acid, methionine. The rest of *c-MYC* sequence until the translation stop codon (TAA) is not shown.

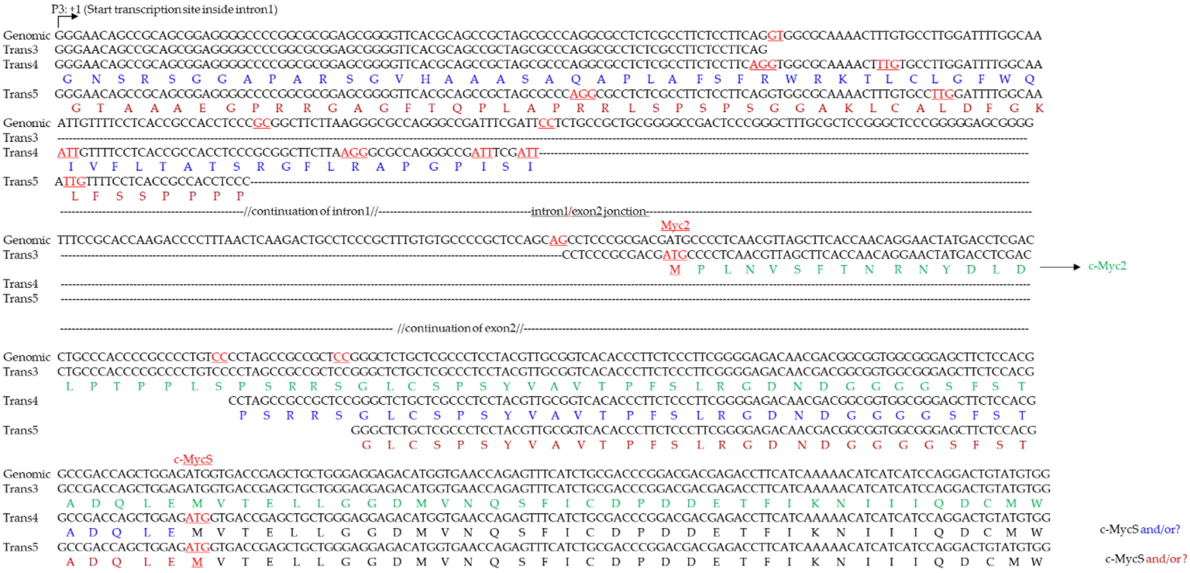

Figure S5: Nucleotide sequences of Trans3, Trans4, and Trans5 transcripts compared to the corresponding genomic structure. Underlined dinucleotides represent the splice sites found. The ATG initiation codons for c-Myc2 and c-MycS are indicated. Trans3 translation predicts c-Myc2 protein. Curiously and despite different splicings, Trans4 and Trans5 transcripts do not have a stop codon in their 5'UTR region. Potential non-ATG initiation sites are underlined. P3:+1: transcription initiation site from the P3 promoter. PFull3: Primer; M: The first amino acid, methionine. The rest of *c-MYC* sequence until the translation stop codon (TAA) is not shown.

```
P?: +1
        Pfull4                    IGH sequence                                                  renaining c-Myc intron1 sequence
BL41    GGAGATAGTGGGGCTCAGAGCATGTCAGAATGTCCTCGGGGAGATCTGTGATATTGAAAGCATTGGTTCACTAAGTGCGTCTCCGAGATAGCAGGGGACTGTCCAAAGGGGGTGAAAGGG 120
Genomic ----------------------------------------------------------------GGTTCACTAAGTGCGTCTCCGAGATAGCAGGGGACTGTCCAAAGGGGGTGAAAGGG 56

BL41    TGCTCCCTTTATTCCCCCACCAAGACCACCCAGCCGCTTTAGGGGATAGCTCTGCAAGGGGAGAGGTTCGGGACTGTGGCGCGCACTGCGCGCTGCGCCAGGTTTCCGCACCAAGACCCC 240
Genomic TGCTCCCTTTATTCCCCCACCAAGACCACCCAGCCGCTTTAGGGGATAGCTCTGCAAGGGGAGAGGTTCGGGACTGTGGCGCGCACTGCGCGCTGCGCCAGGTTTCCGCACCAAGACCCC 176
                                                     c-Myc2          (exon2)
BL41    TTTAACTCAAGACTGCCTCCCGCTTTGTGTGCCCCGCTCCAGCAGCCTCCCGCGACGATGCCCCTCAACGTTAGCTTCACCAACAGGAACTATGACCTCGACTACGACTCGGTGCAGCCG 360
Genomic TTTAACTCAAGACTGCCTCCCGCTTTGTGTGCCCCGCTCCAGCAGCCTCCCGCGACGATGCCCCTCAACGTTAGCTTCACCAACAGGAACTATGACCTCGACTACGACTCGGTGCAGCCG 296
                                                         M  P  L  N  V  S  F  T  N  R  N  Y  D  L  D  Y  D  S  V  Q  P  ------> c-Myc2
```

Figure S6: Nucleotide sequences of the only transcript expressed from a yet to be determined promoter (P?). The transcript exhibits an IGH sequence. This transcript encodes c-Myc2. PFull4: Primer; M: Methionine. The rest of *c-MYC* sequence until the translation stop codon (TAA) is not shown.
